# Risk Factors for Late Diagnosis of Rett Syndrome

**DOI:** 10.15844/pedneurbriefs-29-5-5

**Published:** 2015-05

**Authors:** J. Gordon Millichap

**Affiliations:** 1 Division of Neurology, Ann & Robert H. Lurie Children's Hospital of Chicago, Chicago, IL; 2 Departments of Pediatrics and Neurology, Northwestern University Feinberg School of Medicine, Chicago, IL

**Keywords:** MECP2, Rett Syndrome, Early Diagnosis, Prognosis, Risk Factors

## Abstract

Investigators at Emory University, Atlanta, GA; Stony Brook, New York; University of California, San Diego; and other centers determined the type of physician who makes the Rett syndrome (RTT) diagnosis and identified risk factors for delayed diagnosis.

Investigators at Emory University, Atlanta, GA; Stony Brook, New York; University of California, San Diego; and other centers determined the type of physician who makes the Rett syndrome (RTT) diagnosis and identified risk factors for delayed diagnosis. Among 919 classic and 166 atypical female RTT participants recruited between 2006 and 2014, the median age at diagnosis was 2.7 years (range 2.0-4.1) in classic and 3.8 years (range 2.3-6.9) in atypical RTT. Pediatricians rarely made the diagnosis of classic RTT (5.2%), but the proportion increased since 2006. Odds of a pediatrician making the diagnosis of classical RTT were higher if a child stopped responding to parental interaction, and lower if they had gastroesophageal reflux, specific stereopathies, or if they lost babbling or ability to follow commands. Earlier diagnosis was associated with delay in gross motor skills or finger feeding. Late diagnosis risk factors were delay in fine motor skills, late onset of supportive features (GE reflux, bruxism, breath-holding, hyperventilation, self-abuse) and normal head circumference; 33% with microcephaly before 2.5 years were diagnosed with RTT after the median age of 2.7 years. Age of RTT diagnosis has improved among subspecialists, and pediatricians made the diagnosis of classic RTT more frequently since 2006. [1]

COMMENTARY. Children with Rett syndrome, almost exclusively female, have delayed milestones. Regression occurs after 12 months in >90%, and is followed by phase of recovery or stagnation. A high index of suspicion between ages 6 months to 3 years, and greater awareness of the diagnostic importance of delay in advanced skills should lead to improvement in the age of diagnosis [1]. The absence of some characteristic clinical signs, especially head circumference deceleration, should not lead to delay in diagnosis. In the present study, of 83% who had microcephaly, 19% were not diagnosed until after 4.1 years. Early diagnosis is important in family planning, and emphasis on intervention services.

Epilepsy is a core symptom of Rett syndrome, occurring in 60 – 70% of patients, with uncontrolled seizures in 30% [2]. Mean age of onset of epilepsy is 4.68 +/-3.5 years, the younger age of onset correlating with severity of epilepsy. Various mutations in the MECP2 gene have a different influence on epilepsy, unrelated to the severity of the Rett phenotype. The p.R255X mutation confers an increased risk for epilepsy and severe epilepsy [2]. Attempts to correlate clinical stages of Rett syndrome with epileptic activity in the EEG have mixed results. Some typical symptoms of Rett syndrome (hand stereotypies, vacant spells) are difficult to differentiate from seizures [3]. EEG abnormalities reported in Rett syndrome patients include continuous spike and wave in slow-wave sleep [4] and diffuse paroxysmal alpha activity [5].
